# Improving MPI and hyperthermia performance of superparamagnetic iron oxide nanoparticles through fractional factorial design of experiments†

**DOI:** 10.1039/d4na00378k

**Published:** 2024-07-23

**Authors:** Yanchen Li, Rui Zhang, Roman Barmin, Elena Rama, Max Schoenen, Franziska Schrank, Volkmar Schulz, Ioana Slabu, Fabian Kiessling, Twan Lammers, Roger M. Pallares

**Affiliations:** a Institute for Experimental Molecular Imaging, RWTH Aachen University Hospital Aachen 52074 Germany rmoltopallar@ukaachen.de; b Institute for Applied Medical Engineering, RWTH Aachen University Hospital Aachen 52074 Germany

## Abstract

Superparamagnetic iron oxide nanoparticles (SPIONs) are widely used for biomedical applications, including magnetic particle imaging (MPI) and magnetic hyperthermia. The co-precipitation method is one of the most common synthetic routes to obtain SPIONs, since it is simple and does not require extreme conditions, such as high temperatures. Despite its prevalence, however, the co-precipitation synthesis presents some challenges, most notably the high batch-to-batch variability, as multiple factors can influence nanoparticle growth. In this study, we utilized a fractional factorial design of experiments to identify key factors influencing SPION growth, properties, and performance in MPI and magnetic hyperthermia, namely Fe^3+^ content, pH, temperature, stirring, and atmosphere. Notably, our study unveiled secondary interactions, particularly between temperature and Fe^3+^ content, as well as pH and Fe^3+^ content, for which simultaneous changes of both parameters promoted greater effects than the sum of each factor effect alone, emphasizing the impact of synergistic effects on SPION growth and performance. These findings provide a deeper understanding of the growth mechanism of SPIONs, reconcile discrepancies in the existing literature, and underscore the importance of characterizing secondary interactions to improve the performance of SPIONs for biomedical applications.

## Abstract 2

We utilized a fractional factorial design of experiments to characterize the key factors influencing SPION growth and performance in MPI and magnetic hyperthermia.

## Graphical abstract

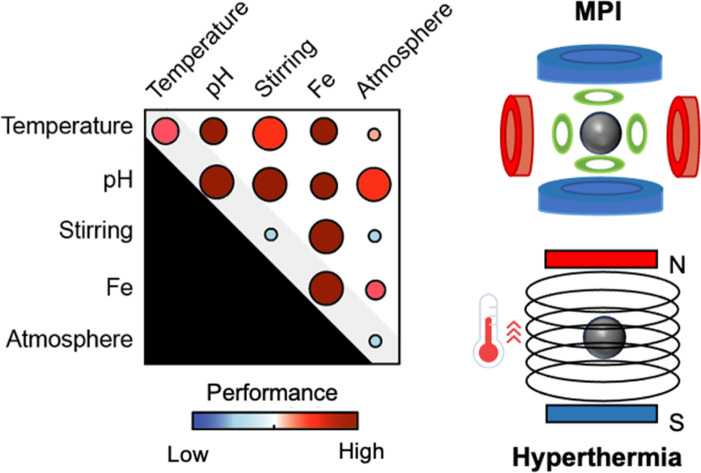

## Introduction

Superparamagnetic iron oxide nanoparticles (SPIONs) are magnetic nanomaterials with unique physicochemical properties that are widely used for (pre)clinical diagnosis and therapy.^1–5^ For example, they display strong magnetic responses when exposed to an external magnetic field, which rapidly decrease when the magnetic field is removed. Their magnetic properties are also exploited in magnetic particle imaging (MPI), an emerging imaging modality that relies on two opposing magnetic gradient fields to saturate the magnetization of SPIONs, except for those located in a field free region that are used as tracers.^6^ Among different (potential) applications, SPIONs have been used as MPI tracers for monitoring immune cell infiltration in pathological tissues during immunotherapy and for real-time imaging of perfusion.^7^ Furthermore, the heat released by SPIONs under the influence of alternating magnetic fields of sufficient strength and frequency can be used to locally ablate pathological tissues, such as tumors, in a therapeutic strategy known as magnetic hyperthermia.^8^ Beyond their magnetic properties, the surface of SPIONs can be easily functionalized with biocompatible ligands and targeting agents, endowing applications in molecular imaging and targeted therapeutics.^9,10^

SPIONs are synthesized through different colloidal methods, including thermal decomposition,^11^ co-precipitation,^12^ sol–gel,^13^ and microemulsion.^14^ The co-precipitation method is frequently used, since it possesses several key advantages, including high yield, simplicity (*e.g.* no high temperatures are required), and production of water-soluble nanoparticles. However, it also presents limitations, such as batch-to-batch variability and poor control over SPION morphology and properties. This variability is likely caused by the effect of several factors (*e.g.* iron content, atmosphere, pH level, temperature, and stirring) that influence the growth of the SPIONs. Notably, previous works with SPIONs obtained through sonication methods and other nanoparticles, such as gold nanorods and gold nanostars, demonstrated that simultaneous changes of several factors during the synthesis of the nanoparticles have synergistic effects, which are greater than the sum of single factor changes.^15–17^ Understanding these multilevel factorial effects is crucial for nanoparticle growth and reducing synthesis variability.

**Scheme 1:**
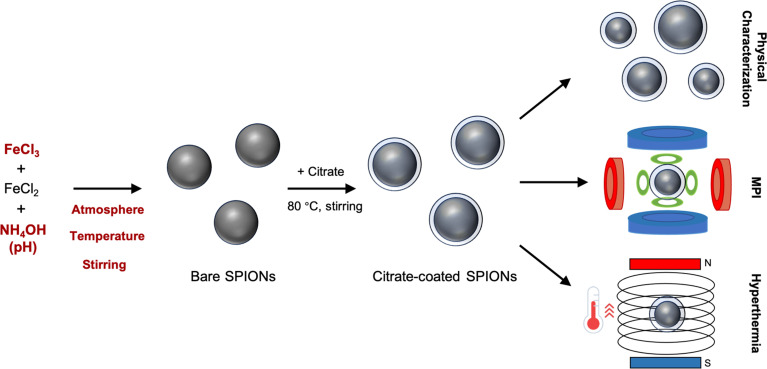
Experimental design of the study. The five key factors (highlighted in red) governing the synthesis and performance of SPIONs were studied through a fractional factorial design of experiments. After the synthesis, the morphological characteristics of the SPIONs as well as their performance in MPI and magnetic hyperthermia were evaluated.

Here, we investigated the primary and secondary interaction effects governing the synthesis and performance of SPIONs through a fractional factorial design of experiments (Scheme 1). The synthesized SPIONs exhibited a nearly spherical shape. Both the size of SPIONs and the signal-to-noise ratio (SNR) in MPI increased with changes in temperature, Fe^3+^ content, and pH. However, the performance of SPIONs in magnetic hyperthermia was found to be less sensitive to the evaluated synthesis factors. Additionally, specific secondary interactions exerted greater effects than the sum of each individual factor effect. These findings offer new insights into the synthesis and performance of SPIONs, elucidating discrepancies in the literature concerning the impact of these specific factors.

## Results and discussion

The synthesis of SPIONs *via* the co-precipitation method is commonly performed as follows:^18^ ferric chloride (8 mmol) and ferrous chloride (4 mmol) are mixed in deionized water, and the pH of the resulting solution is adjusted to 11. This mixture is left to react for 30 min at 25 °C under stirring in a nitrogen atmosphere. Subsequently, black iron oxide particles are formed, which are then magnetically separated, washed, and surface functionalized with trisodium citrate (0.25 g mL^−1^) at 80 °C for 2 h. The resulting citrate-coated SPIONs are then magnetically separated, resuspended in deionized water, and filtered with a 0.2 μm filter to remove larger particulates. It is worth noting that SPIONs need to be functionalized (*e.g.* with dextran or PEG) to be stable in biological environments and be used either as therapeutic agents or imaging probes. In our current study, we have focused on their synthesis. Hence, the surface of the SPIONs is modified with citrate, which renders disperse nanoparticles in solution, but not in biological media. Our SPIONs would need to be further functionalized to be (pre)clinically used.

**Fig. 1:**
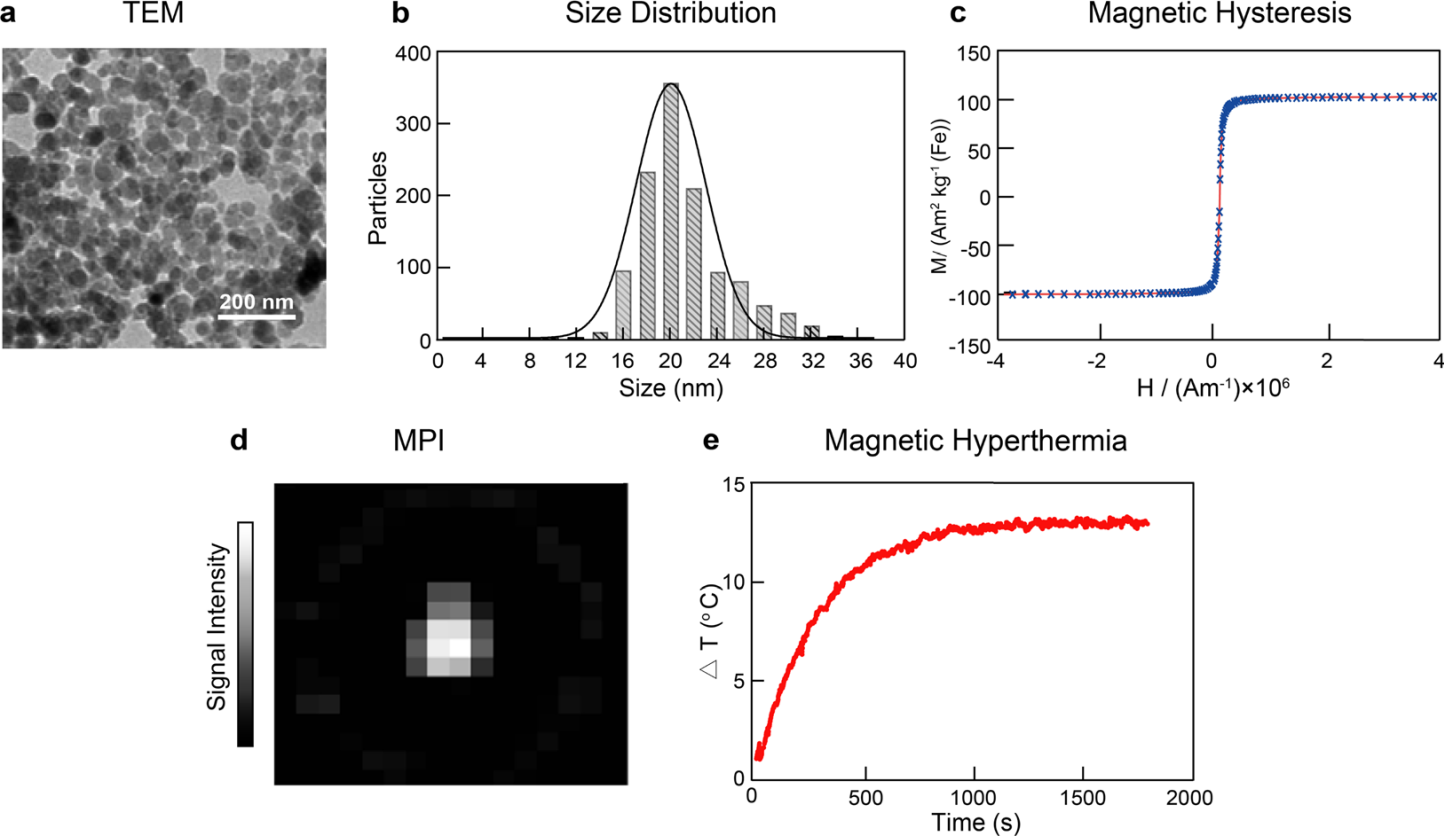
SPIONs obtained with the standard co-precipitation method. (a) TEM micrographs of SPIONs. (b) Size distribution of SPIONs. (c) Field-dependent magnetization curve of SPIONs (2.8 mg mL^−1^) at 295 K. (d) MPI intensity map of SPION solution (0.8 μL, 20.1 mg mL^−1^). (e) Time–temperature curves of SPIONs (1 mg mL^−1^) under the influence of an alternating magnetic field at a frequency of 271 kHz and a field amplitude of 41 kA m^−1^ for 30 min.

After performing the synthesis under these conditions, we obtained nanoparticles with spheroid-like shape and an average core diameter of 21.2 ± 3.8 nm, as observed by transmission electron microscopy (TEM) (Fig. 1a and b). The SPIONs displayed superparamagnetic behavior as identified in the hysteretic *M*(*H*) curves at 295 K, with an average saturation magnetization of 101.8 ± 0.7 A m^2^ kg^−1^, indicating strong magnetic responses to the external magnetic field (Fig. 1c). Furthermore, the synthesized SPIONs showed good imaging capabilities by generating intense signals in MPI (Fig. 1d). The SPIONs were also able to increase the temperature of the solution by 13.1 ± 0.2 °C when placed under the influence of an alternating magnetic field (frequency of 271 kHz and amplitude of 41 kA m^−1^) in a custom-built hyperthermia setup (Fig. 1e).

In the current study, five key synthetic parameters were investigated through fractional factorial design of experiments (Table 1). These five factors were chosen based on previous reports, which identified them as parameters influencing the growth of SPIONs.^18,19^ Because the molar ratio of ferric chloride and ferrous chloride was kept constant at 2 : 1, only the variation of Fe^3+^ is shown in the table. Additionally, each factor was examined at two different levels. Hence, the resulting experimental design, known as 2_V_^5-1^, was comprised of 5 factors with 2 levels each, involving a total of 16 experiments (Table S1†). Notably, the 2_V_^5-1^ design enables the identification of both primary and secondary interaction effects.^20,21^ The morphological characteristics of the SPIONs grown under each condition were characterized by TEM, and their performances as MPI tracers, and magnetic hyperthermia agents were also assessed.

**Table 1:** Experimental conditions used for each factor, and their comparison to the standard protocol

	Factors	Standard protocol	Low (−1)	High (+1)
A	Temperature	25 °C	25 °C	80 °C
B	pH	11	9	11
C	Fe^3+^	8 mmol	8 mmol	40 mmol
D	Atmosphere	N_2_	Air	N_2_
E	Stirring	100 rpm	0	100 rpm

### Size of the SPIONs

The nanoparticles were synthesized under the different conditions reported in Table S1† in quadruplicate. Out of the sixteen conditions explored, four conditions (FFD 2, 6, 10 and 14 in Table S1†) with Fe^3+^ content of 40 mmol and pH of 9 did not yield SPIONs. This observation implies that SPIONs cannot be generated when the iron content is high and the pH is low simultaneously. In contrast, the rest of the conditions did allow the growth of SPIONs, which were characterized by TEM (Fig. S1 and S2†) and displayed inter-batch variabilities between 2.4 and 6.8% (Table S2†). We initially characterized the effect of the five factors on the diameter of the SPIONs. Since *t*-tests tend to overestimate statistical significance when comparing large populations (in our case, between hundreds and thousands of nanoparticles per experimental group), we employed effect size (Cohen's *d*) to determine the strength of an effect when comparing two populations of sizes.^22^ Further details on the statistical analysis can be found in the materials and methods section.

**Fig. 2:**
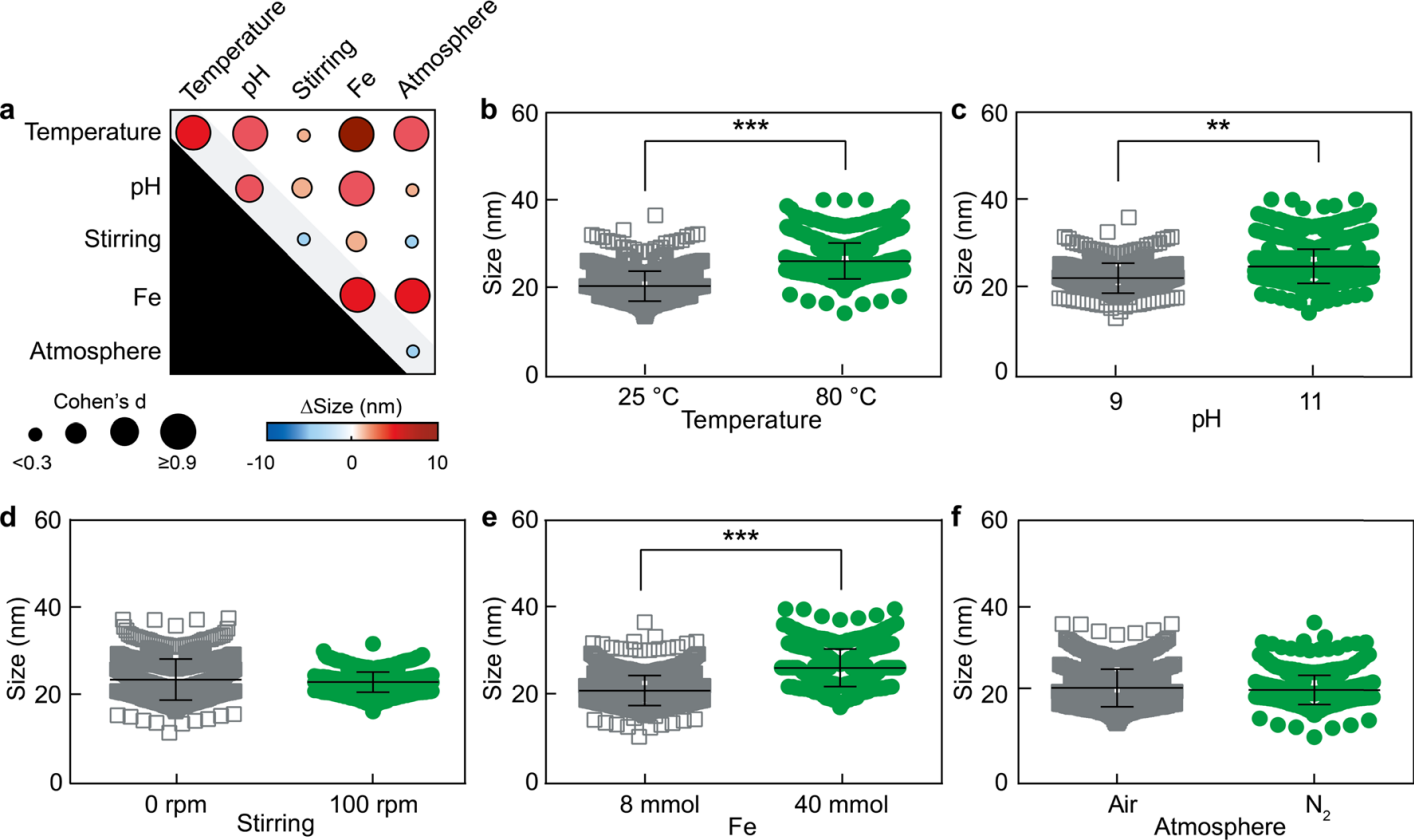
Primary and secondary interaction effects on the SPION size. (a) Summary of the interaction effects, and primary interaction results of (b) temperature, (c) pH, (d) stirring, (e) Fe^3+^ content, and (f) atmosphere on SPION size (diameter). (**) and (***) indicate groups with large (Cohen's *d* > 0.6) and very large (Cohen's *d* > 0.9) effect sizes, respectively. Each data point represents the size of a measured particle. Four batches per condition were synthesized and more than 100 particles were measured per batch.

The fractional factorial design showed that primary and secondary interaction effects of three factors determine the size of SPIONs (Fig. 2). These three key factors are temperature, Fe^3+^ content, and pH value (Cohen's *d* > 0.3). The statistical analysis of secondary interaction effects is displayed in Fig. S3.† It must be noted that the experimental data show a wide dispersion when presented as function of the primary interaction effects. This is because the data are displayed based on only one factor (*e.g.*, low or high Fe^3+^ content), even though the values of the other four factors (*e.g.*, temperature, pH, stirring, and atmosphere) may be different depending on the experimental conditions set during the synthesis of each SPION batch (Table 1). Nevertheless, the 2_V_^5-1^ fractional factorial design can discriminate primary and secondary interaction effects.^20,21^

Our analysis demonstrated that Fe^3+^ content was the most significant factor, through both primary and several secondary interaction effects, in influencing the size of SPIONs (Cohen's *d* > 0.9). As Fe^3+^ content increased from low (8 mmol) to high (40 mmol), the diameter of the SPIONs increased by 4.1 ± 0.2 nm. Additionally, secondary interaction effects of Fe^3+^ content could also increase the SPION diameter from 18.8 ± 2.2 nm up to 24.3 ± 5.0 nm. The higher iron ion content endowed the growth of larger particles, likely because of the greater availability of iron in solution to be incorporated into the nanoparticles.

Moreover, an increase in solution pH also correlated with an augmented SPION diameter, influenced by both primary and secondary interaction effects (from 19.7 ± 2.0 up to 24.2 ± 3.4 nm). Although the mechanism behind this observation is not fully understood, it is worth noting that increasing the pH from 9 to 11 promotes the formation of soluble iron hydroxide species (*i.e.* Fe(OH)_4_^−^)^23^ that may favor the formation of larger particles through different colloidal mechanisms, such as Ostwald ripening.^24,25^

The third factor to affect SPION size was temperature. Increasing the temperature from 25 °C to 80 °C, led to an increase in particle size up to 26.1 ± 3.3 nm, likely due to the faster nanoparticle growth kinetics at higher temperatures, which are known to induce larger particulates through different mechanisms, such coalescence and Ostwald ripening.^24,26^ Our quantitative results on the primary effects of temperature, Fe^3+^ content, and pH on SPION size align with qualitative observations previously published.^27^

Notably, the most significant change in SPION diameter (increasing from 19.9 ± 3.8 nm to 28.3 ± 5.1 nm) was caused by the secondary interaction between temperature and Fe^3+^ content. This secondary interaction effect caused a greater size variation than the sum of each primary effect alone, demonstrating the importance of secondary interactions during the synthesis and growth of SPIONs.

### SNR measured by MPI

MPI is an advanced imaging technique known for its high temporal and spatial resolution, allowing real-time 3D visualization of SPION distribution, providing valuable information on their localization and concentration.^28–31^ SNR is a critical metric in MPI, as a higher SNR enhances the quality and reliability of the images by minimizing unwanted noise and maximizing the clarity of the signal.^32^

**Fig. 3:**
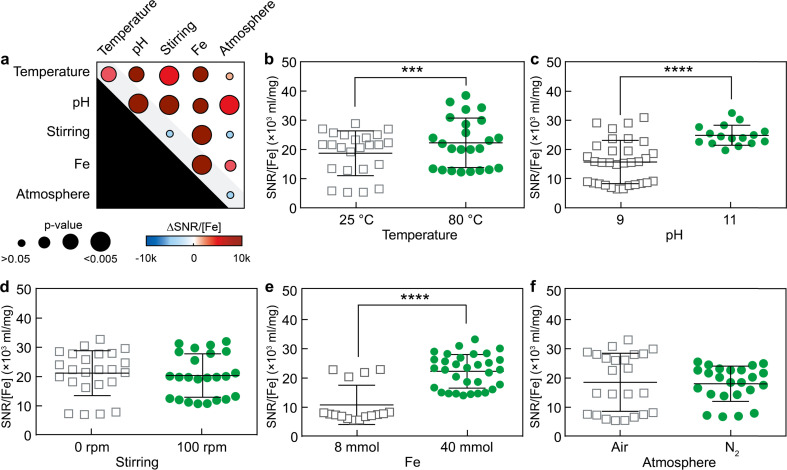
Primary and secondary interaction effects on the SNR as measured by MPI. (a) Summary of the interaction effects, and primary interaction results of (b) temperature, (c) pH, (d) stirring, (e) Fe^3+^ content, and (f) atmosphere on SNR of SPIONs as measured by MPI. (***) and (****) indicate groups that are significantly different with *p* < 0.001, and *p* < 0.0001, respectively (independent two-sample *t*-test). Each data point represents the SNR of a batch solution. Four batches were tested per condition.

Therefore, the primary and secondary effects of the five factors on the SNR of SPIONs during MPI measurements were characterized. To compare the MPI performance of the various samples, SNR values were normalized to the iron concentration of each sample. Both primary and secondary interaction effects, including variations in Fe^3+^ content, pH, and temperature, were found to statistically influence the magnitude of SNR measured by MPI (Fig. 3). These observations were consistent with the results obtained for size variations in Fig. 2. For example, Fe^3+^ content was the strongest factor affecting SPION diameter, increasing nanoparticle size through primary and secondary interaction effects. Consistently, as Fe^3+^ content increased from low (8 mmol) to high (40 mmol) values, the SNR strength escalated from 10.6 ± 1.7 × 10^3^ to 22.1 ± 1.1 × 10^3^.

Moreover, varying the pH from 9 to 11 and the temperature from 25 °C to 80 °C increased the SNR by 2.9 ± 2.3 × 10^3^ and 8.7 ± 1.6 × 10^3^, respectively. The statistical analysis of secondary interaction effects on SNR is reported in Fig. S4.† Notably, two secondary interactions, namely pH-stirring and Fe-stirring, caused the most pronounced effects on the SNR as measured by MPI, increasing the SNR up to 26.8 ± 1.4 × 10^3^ and 24.8 ± 1.3 × 10^3^, respectively. This is puzzling because variations of stirring alone have almost no discernible impact on SNR, and the primary effects of pH or iron content are notably less pronounced than those observed in the context of pH-stirring or Fe-stirring. These results further highlighted the importance of secondary effects, which can be larger than the sum of the effects of each factor alone.

### Temperature changes observed during magnetic hyperthermia

When SPIONs are placed in a alternating magnetic field on the order of a few hundred kHz, their magnetic moments continuously reorient following the field direction, which causes the local release of heat due to relaxation losses.^33^ This principle has been extensively explored in preclinical settings for local thermal ablation of pathological tissue (magnetic hyperthermia) in treatments against different types of tumors, and is currently being explored in a clinical trial against advanced pancreatic cancer.^34^

The evaluation of heating performance involves the measurement of the specific loss power (SLP), which represents the thermal loss per unit mass under the influence of an applied magnetic field.^35,36^ To assess SLP values, we conducted temperature–time curve measurements for each sample at a concentration of 1 mg mL^−1^ under the influence of an alternating magnetic field (frequency of 271 kHz and amplitude of 41 kA m^−1^), as depicted in Fig. S5.† Further details on the SLP calculations can be found in the Materials and methods section.

**Fig. 4:**
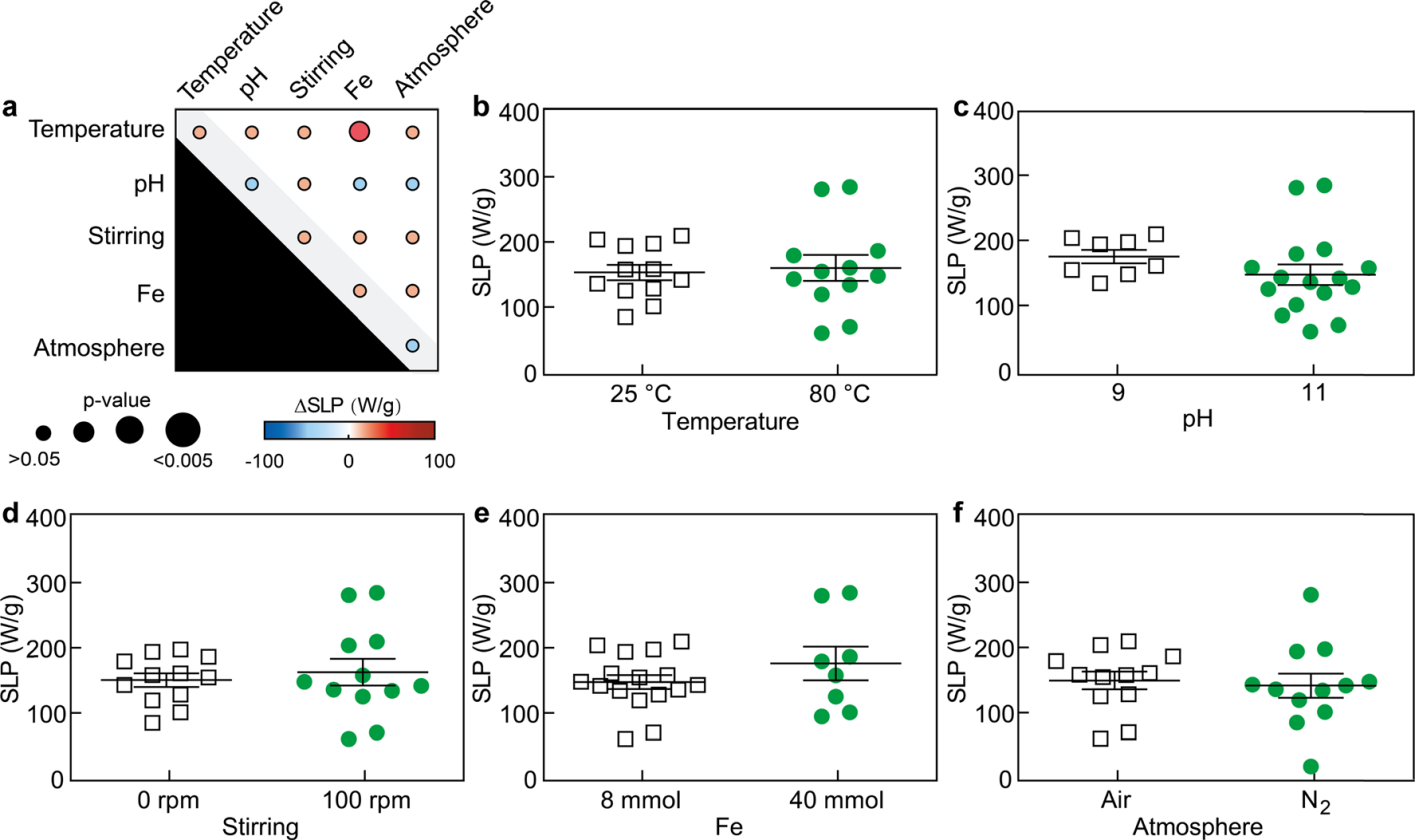
Primary and secondary interaction effects on the SLP. (a) Summary of the interaction effects, and primary interaction results of (b) temperature, (c) pH, (d) stirring, (e) Fe^3+^ content, and (f) atmosphere on SPION performance in magnetic hyperthermia. Each data point represents the SLP of a batch solution. Two batches were tested per condition.

By characterizing the primary and secondary effects of the five factors on the magnetic hyperthermia performance of the resulting SPIONs, we observed that SLP values were barely statistically affected by either primary or secondary interaction effects (Fig. 4 and S6†). As previously discussed, the data show large variability within groups when displayed as function of the primary interaction effects, since the results are presented based on one factor (*e.g.*, low or high pH) independently of the values of the other four factors (*e.g.*, temperature, Fe^3+^ content, stirring, and atmosphere) set during the experiment. Only the Fe^3+^ content – temperature effects did statistically alter (increased) the SLP value (*p* < 0.05). These results seem to suggest the performance of SPIONs for magnetic hyperthermia is less sensitive to the synthetic factors evaluated, and perhaps, stronger variations of those factors would be necessary to cause changes in their SLP and further improve their performance.

## Conclusion

In summary, we employed a fractional factorial design of experiments to unravel the key factors influencing the co-precipitation synthesis of SPIONs and their performance in MPI, and magnetic hyperthermia. Fe^3+^ content, pH, and temperature, either individually or particularly in combination with other factors, were found to impact the size of SPIONs the most. Notably, changes in stirring alone had minimal effects but exhibited a significant impact on the SNR of MPI when combined with other factors. Magnetic hyperthermia was the application less affected by changes in the five factors, and only simultaneous variations of temperature and iron content increased the SLP of the particles. These findings underscore the intricate nature of the factors governing the growth of SPIONs and emphasize the importance of characterizing secondary interaction effects for tailoring nanoparticles with desired properties to specific applications.

## Data availability

The data supporting this article have been included as part of the ESI.†

## Author contributions

Y. L. and R. M. P. designed the research; Y. L. carried out the synthesis and characterization of the nanoparticles; R. Z., R. B. and E. R. assisted in the experiments; M. S. and I. S. performed the magnetic hyperthermia tests; F. S. carried out the MPI experiments; V. S., F. K., T. L. and R. M. P. provided scientific guidance; Y. L. wrote the first manuscript draft; all authors reviewed the manuscript.

## Conflicts of interest

The authors have no relevant affiliations or financial involvement with any organization or entity with a financial interest in or financial conflict with the subject matter or materials discussed in the manuscript.

## Supplementary Material

NA-006-D4NA00378K-s001
