# Supplementary material for: Improving MPI and hyperthermia performance of superparamagnetic iron oxide nanoparticles through fractional factorial design of experiments
Source: Nanoscale Adv. 2024 Jul 23;6(17):4352–9. doi: 10.1039/d4na00378k (PMC11334983; doi:10.1039/d4na00378k)
Supplement: NA-006-D4NA00378K-s001 [file NA-006-D4NA00378K-s001.pdf]

Electronic Supplementary Material (ESI) for Nanoscale Advances.
This journal is © The Royal Society of Chemistry 2024

- SUPPORTING INFORMATION -

Improving MPI and Hyperthermia Performance of Superparamagnetic Iron Oxide Nanoparticles through Fractional Factorial Design of Experiments

Yanchen Li,[1] Rui Zhang,[1] Roman Barmin,[1] Elena Rama,[1] Max Schoenen,[2] Franziska Schrank,[1] Volkmar Schulz,[1] Ioana Slabu,[2] Fabian Kiessling,[1] Twan Lammers,[1] and Roger M. Pallares[1,*]

[1]Institute for Experimental Molecular Imaging, RWTH Aachen University Hospital, Aachen 52074, Germany

[2]Institute for Applied Medical Engineering, RWTH Aachen University Hospital, Aachen 52074, Germany

[*]Corresponding author: rmoltopallar@ukaachen.de

Table of Contents

Materials and methods S2

Table S1. Fractional factorial design of experiments. S3

Figure S1. TEM micrographs of synthesized SPIONs. S6

Figure S2. Size distributions of the SPIONs.. S7

Table S2. Average SPION size and inter-batch coefficient of variation. S8

Figure S3. Secondary interaction effects on S9

Figure S4. Secondary interaction effects on S10

Figure S5. Magnetic hyperthermia performance of SPIONs.. S11

Figure S6. Secondary interaction effects on the SLP of SPIONs. S12

Materials and methods

Materials

Ferric chloride anhydrous and ferrous chloride tetrahydrate were purchased from Sigma-Aldrich (Munich, Germany). Trisodium citrate dihydrate, hydrochloric acid and ammonium hydroxide were purchased from Carl Roth (Karlsruhe, Germany). Deionized (DI) water was produced by a PURELAB flex 2 device from ELGA LabWater (Celle, Germany) and used for all experiments. All reagents were of appropriate analytical grade.

Super paramagnetic iron oxide nanoparticle (SPION) synthesis and fractional factorial design of experiments

The general synthesis of SPIONs was based on a previously reported method and it was performed in quadruplicate.[1] Briefly, at 25 °C, 8 mmol of ferric chloride was dissolved in deionized water, followed by the addition of 4 mmol of ferrous chloride tetrahydrate. The pH was adjusted to 11.0 using 1 M aqueous ammonia solution, and the solution was stirred for 30 min in a nitrogen atmosphere. The total volume of the solution was 92 mL and the synthesis was carried out in a 500 mL three-neck round-bottom flask. The resulting black iron oxide particles were separated using a permanent magnet and washed thrice with DI water. Subsequently, 20 mL hydrochloric acid (0.1 M) was added to the particles and sonicated for 10 min. After sonication, 10 mL citrate solution (final concentration of 0.25 g/mL) was added, and the mixture was stirred at 80 °C for 2 h. Finally, the suspension was passed through a 0.2 µm filter to remove the large particulates and resuspended in 35 mL of DI water.

For the synthesis of the SPIONs under different conditions, the same protocol was followed, replacing specific conditions (Fe^{3+} content, pH, temperature, stirring and atmosphere during the nanoparticle growth) as defined in Tables 1 and S1. The synthesis for each condition was performed in quadruplicate. The surface of all SPIONs was modified with citrate as described in the standard protocol.

Table S1. Fractional factorial design of experiments.					
Experiment	Fe^{3+}	pH	Temperature	Stirring	Atmosphere
FFD 1	-1	-1	-1	-1	+1
FFD 2	+1	-1	-1	-1	-1
FFD 3	-1	+1	-1	-1	-1
FFD 4	+1	+1	-1	-1	+1
FFD 5	-1	-1	+1	-1	-1
FFD 6	+1	-1	+1	-1	+1
FFD 7	-1	+1	+1	-1	+1
FFD 8	+1	+1	+1	-1	-1
FFD 9	-1	-1	-1	+1	-1
FFD 10	+1	-1	-1	+1	+1
FFD 11	-1	+1	-1	+1	+1
FFD 12	+1	+1	-1	+1	-1
FFD 13	-1	-1	+1	+1	+1
FFD 14	+1	-1	+1	+1	-1
FFD 15	-1	+1	+1	+1	-1
FFD 16	+1	+1	+1	+1	+1

Transmission electron microscopy (TEM)

TEM was employed to determine the size (diameter) of SPIONs. Each SPION solution was washed by centrifugation (9,000 rpm for 10 min) and re-suspended in DI water before being drop casted on a 200-mesh carbon filmed copper grid (Plano GmbH, Wetzlar, Germany). Grids were dried at room temperature overnight before being imaged with a Hitachi TEM system (Hitachi, Ibaraki, Japan) at 100 kV.

Magnetization characterization

The samples were magnetically characterized using a SQUID magnetometer MPMS 5S (LOT Quantum Design, USA). For the measurements, the particles were dispersed in a 13 wt % mannitol solution and freeze dried at -85 °C and 0.04 mbar in a lyophilisator (Alpha 2-4 LDplus, Germany) for 24 h.

Magnetization measurements were performed at 295 K varying the field strength from zero to 4×10^6 A/m. The saturation magnetization MS was determined by fitting the Langevin function

$$L(\xi) = coth(\xi) - \frac{1}{\xi}$$

to the measured data. Here, $\xi = \mu H/(k_B T)$ and $\mu = V_M M_S$ denotes the particle magnetic moment with V_M the mean magnetic volume, H the applied magnetic field and $k_B = 1.38 \times 10^{-23}$ J/K the Boltzmann constant.

Magnetic particle imaging (MPI)

MPI experiments were conducted on a pre-clinical MPI system (Bruker Biospin GmbH, Ettlingen, Germany) using a dedicated x-receive coil for mice. The static selection field featured a gradient of 2.5 mT/µ0 in the steepest direction. For three dimensional MPI data, three orthogonal dynamic drive fields with amplitudes of 14 mT/µ0 were operated at frequencies fx = 24.51 kHz, fy = 26.04 kHz, and fz = 25.25 kHz. Spectroscopic one-dimensional MPI measurements utilized an excitation field strength of 14 mT/µ0 at frequency fx.

The signal-to-noise ratio (SNR) parameter was calculated as defined by the equation:

$$\text{SNR} = \frac{P_{single}}{P_{noise}}$$

Here, P_{signal} represents the power of the background-corrected sample signal, and P_{noise} is the power of background noise. The SNR was determined through magnetic particle spectroscopy (MPS) measurements. A small vial, containing 8 µL of sample, was positioned at the isocenter of the MPI device, with one thousand repetitions acquired for averaging.

Magnetic hyperthermia

Heating measurements were conducted using a custom-built hyperthermia setup (Trumpf Hüttinger, Freiburg, Germany). Each sample underwent exposure to an alternating magnetic field (AMF) at a frequency of 271 kHz and a field amplitude of 41 kA/m for 30 min. The sample temperature was continuously monitored during AMF exposure using a fiber-optic thermometer Luxtron 812 (LumaSense Inc., Santa Barbara, CA, USA). Background

subtraction was performed utilizing data from reference measurements. The reference sample comprises deionized water.

The specific loss power (SLP) was calculated with the expression SLP = c/ρ $\cdot \frac{dT}{dt}_{t\to 0}$, incorporating the specific heat capacity of water c = 4.187 J/(g·K), the weight fraction of magnetic nanoparticles (ρ), and the initial temperature rise $dT/dt_{t\to 0} = T_{rise} \cdot b$. The Box-Lucas function T(t) = $T_{rise} \cdot (1 - \exp(-b \cdot t)) + T_0$ was fitted to the temperature data. The iron concentration was 1 mg/mL for every sample.

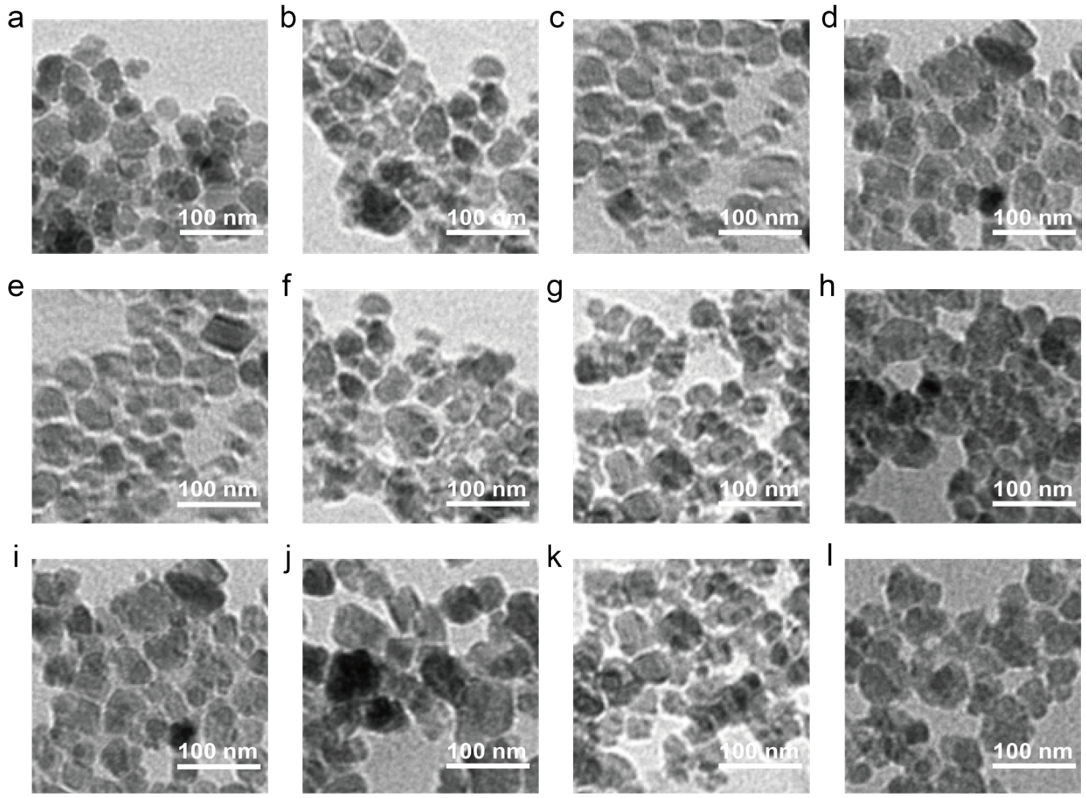

Figure S1. TEM micrographs of synthesized SPIONs. The SPIONs were obtained under the different conditions of the fractional factorial design in Table S1: (**a**) FFD 1, (**b**) FFD 3, (**c**) FFD 4, (**d**) FFD 5, (**e**) FFD 7, (**f**) FFD 8, (**g**) FFD 9, (**h**) FFD 11, (**i**) FFD 12, (**j**) FFD 13, (**k**) FFD 15, (**l**) FFD 16. The experimental conditions FFD 2, FFD 6, FFD 10, and FFD 14 did not produce SPIONs.

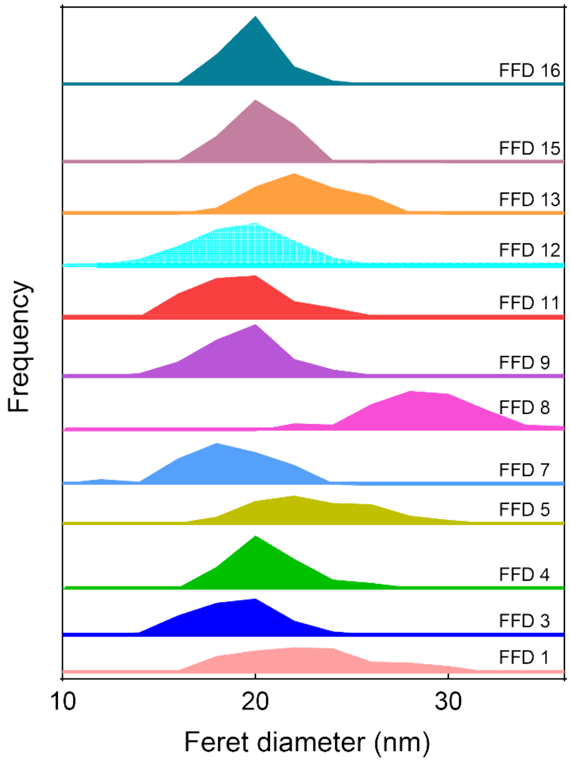

Figure S2. Size distributions of the SPIONs. The size of the SPIONs is reported as their Feret diameter.

Table S2. Average SPION size and inter-batch coefficient of variation.

	Batch 1 (nm)		Batch 2 (nm)		Batch 3 (nm)		Batch 4 (nm)		Coefficient of variation (%)
	Average	Standard deviation	Average	Standard deviation	Average	Standard deviation	Average	Standard deviation	
FFD 1	25.3	3.5	25.8	0.7	24.7	1.0	26.1	2.0	2.4
FFD 3	17.8	1.6	18.6	1.7	17.8	1.6	16.8	1.6	4.2
FFD 4	20.8	2.3	20.5	1.9	21.7	1.8	22.8	2.4	4.8
FFD 5	23.0	2.9	23.9	2.9	22.9	2.9	22.2	2.5	3.0
FFD 7	18.3	2.4	20.3	2.4	19.3	2.4	19.9	2.5	4.5
FFD 8	28.7	3.0	27.7	3.1	27.0	1.0	29.7	3.0	4.2
FFD 9	19.4	2.2	21.9	2.3	20.9	2.3	19.9	2.3	5.4
FFD 11	19.2	2.4	20.5	3.9	18.7	2.3	19.6	2.3	3.9
FFD 12	19.2	2.4	21.4	3.7	19.2	2.4	18.9	2.4	5.9
FFD 13	22.4	2.1	23.6	1.8	20.5	1.36	23.9	1.7	6.8
FFD 15	20.4	1.6	21.1	1.9	20.3	1.3	19.1	1.3	4.1
FFD 16	19.9	1.4	19.6	1.3	20.1	1.4	18.8	1.4	2.9

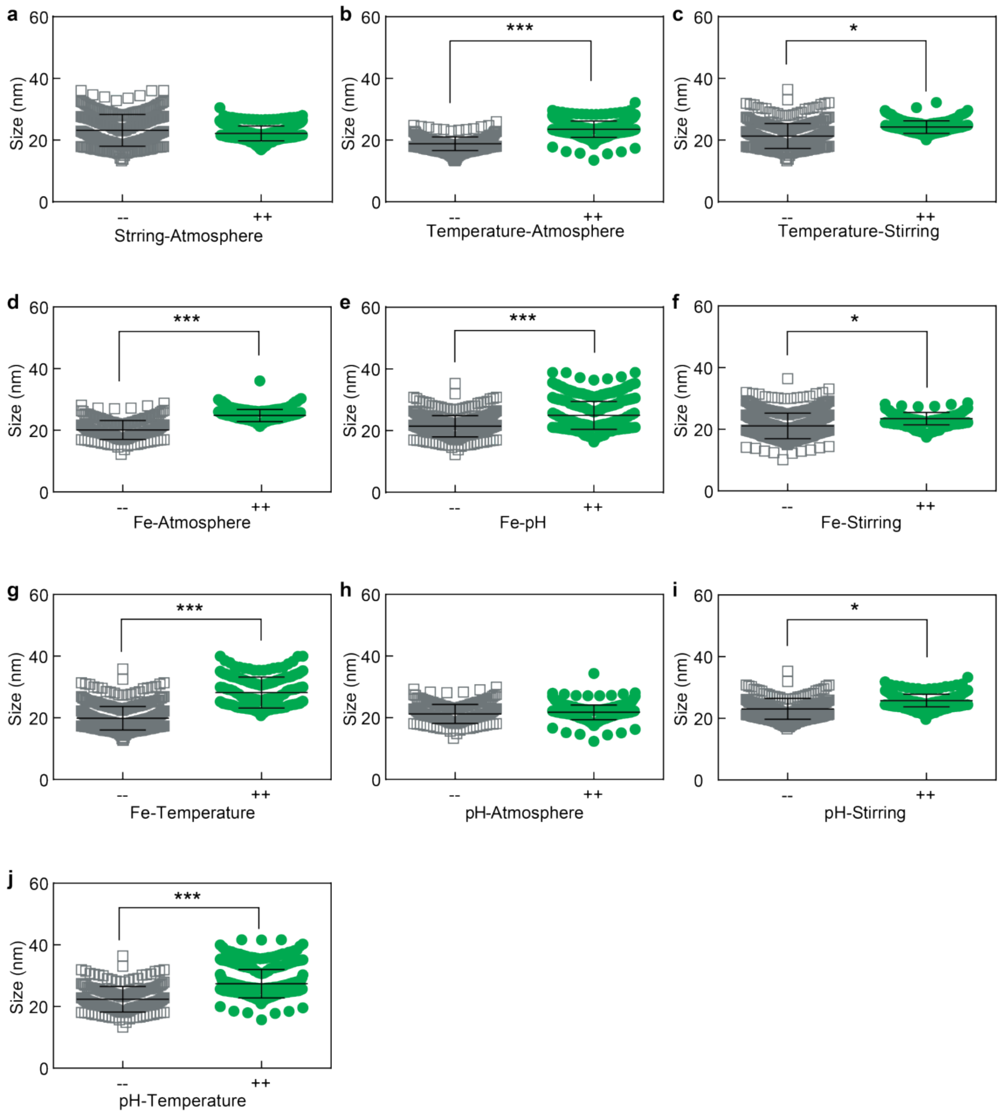

Figure S3. Secondary interaction effects on the SPION size. Secondary interaction effects of (**a**) stirring−atmosphere, (**b**) temperature−atmosphere, (**c**) temperature−stirring, (**d**) Fe^{3+} content−atmosphere, (**e**) Fe^{3+} content−pH, (**f**) Fe^{3+} content−stirring, (**g**) Fe^{3+} content−temperature, (**h**) pH−atmosphere, (**i**) pH−stirring, (**j**) pH−temperature on the SPIONs size. (*) and (***) indicate groups with medium (Cohen's d > 0.3) and very large (Cohen's d > 0.9) effect sizes, respectively.

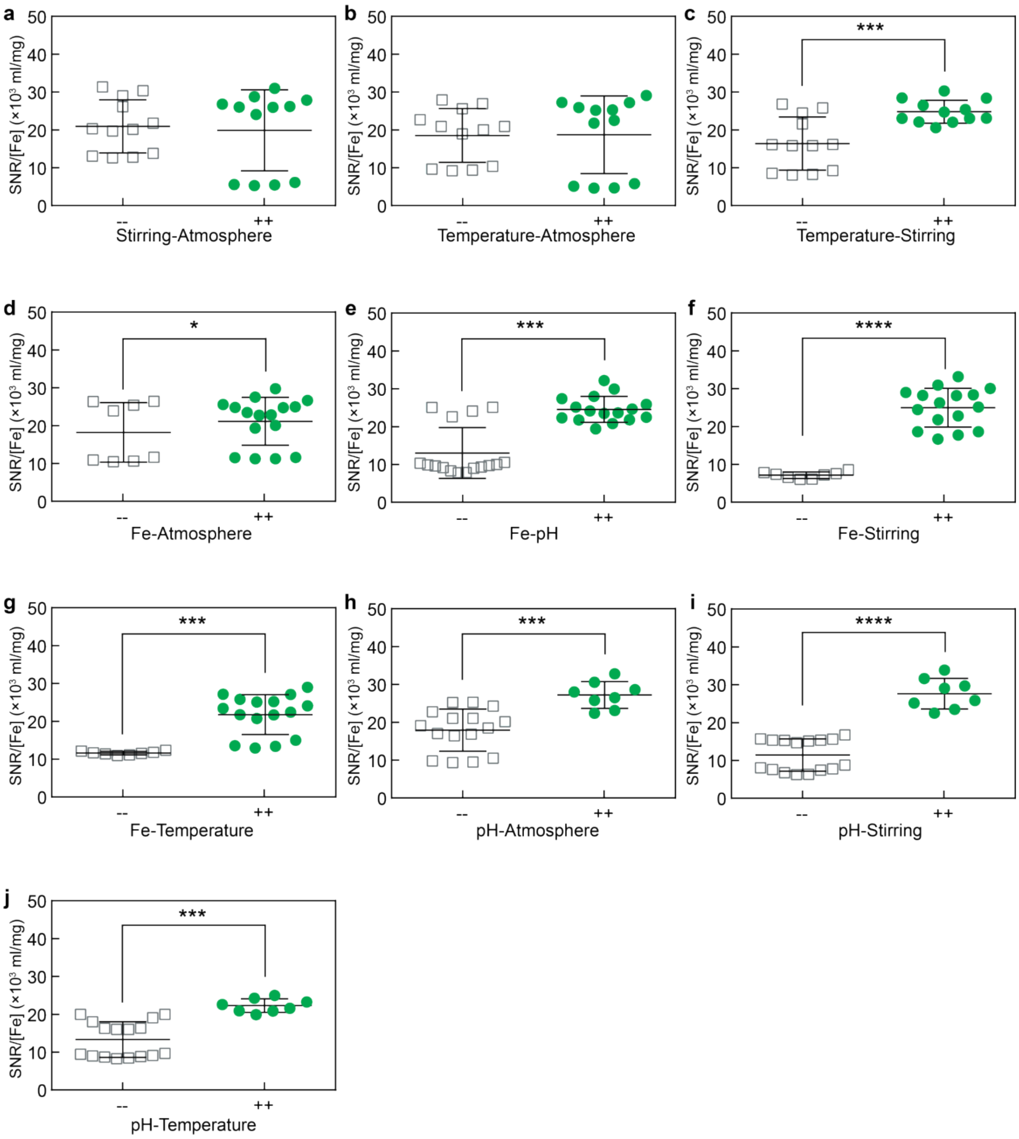

Figure S4. Secondary interaction effects on the SNR as measured by MPI. Secondary interaction effects of (**a**) stirring–atmosphere, (**b**) temperature–atmosphere, (**c**) temperature–stirring, (**d**) Fe^{3+} content–atmosphere, (**e**) Fe^{3+} content–pH, (**f**) Fe^{3+} content–stirring, (**g**) Fe^{3+} content–temperature, (**h**) pH–atmosphere, (**i**) pH–stirring, (**j**) pH–temperature on the SNR. (*), (***) and (****) indicate groups that are significantly different with $p < 0.05$, $p < 0.001$ and $p < 0.0001$, respectively (independent two-sample *t*-test).

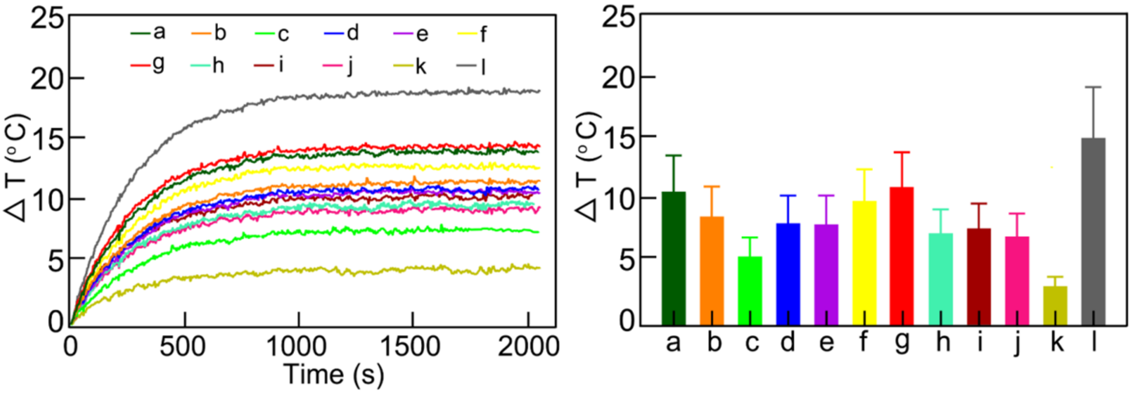

Figure S5. Magnetic hyperthermia performance of SPIONs. Representative temperature – time curves (left) and maximum increase of temperature (right) of SPIONs from the fractional factorial design in Table S1: (a) FFD 1, (b) FFD 3, (c) FFD 4, (d) FFD 5, (e) FFD 7, (f) FFD 8, (g) FFD 9, (h) FFD 11, (i) FFD 12, (j) FFD 13, (k) FFD 15, (l) FFD 16.

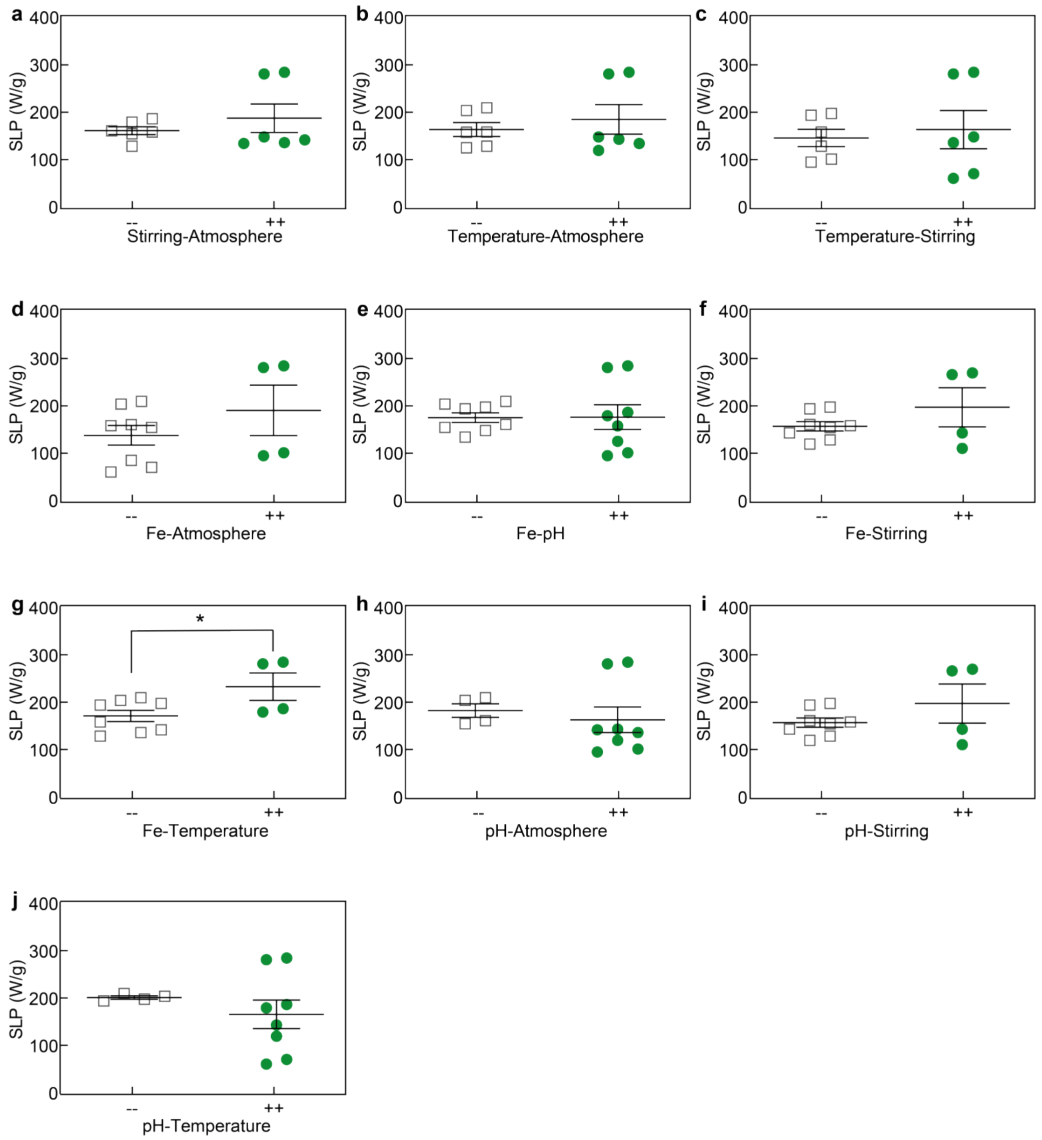

Figure S6. Secondary interaction effects on the SLP of SPIONs. Secondary interaction effects of (**a**) stirring−atmosphere, (**b**) temperature−atmosphere, (**c**) temperature−stirring, (**d**) Fe^{3+} content−atmosphere, (**e**) Fe^{3+} content−pH, (**f**) Fe^{3+} content−stirring, (**g**) Fe^{3+} content−temperature, (**h**) pH−atmosphere, (**i**) pH−stirring, (**j**) pH− temperature on the SLP. (*) indicate groups that are significantly different with $p < 0.05$ (independent two-sample t-test).

References

(1) Dadfar, S. M.; Camozzi, D.; Darguzyte, M.; Roemhild, K.; Varvarà, P.; Metselaar, J.; Banala, S.; Straub, M.; Güvener, N.; Engelmann, U.; Slabu, I.; Buhl, M.; Van Leusen, J.; Kögerler, P.; Hermanns-Sachweh, B.; Schulz, V.; Kiessling, F.; Lammers, T. Size-Isolation of Superparamagnetic Iron Oxide Nanoparticles Improves MRI, MPI and Hyperthermia Performance. J Nanobiotechnol 2020, 18 (1), 22.

(2) Gremse, F.; Stärk, M.; Ehling, J.; Menzel, J. R.; Lammers, T.; Kiessling, F. Imalytics Preclinical: Interactive Analysis of Biomedical Volume Data. Theranostics 2016, 6 (3), 328–341.
